# An Urticaria Appearing Annually

**Published:** 1884-09

**Authors:** J. T. Kent

**Affiliations:** St. Louis


### AN URTICARIA APPEARING ANNUALLY.

Prof. J. T. Kent, A. M., M. D., St. Louis.

Mrs. S., about forty years old, wife of a prominent clergyman in this city, consulted me for annually appearing paroxysms of urticaria, or whatever you may be pleased to name it. On the 13th of May every year for seven years she had been seized with a burning and itching of the skin that would seem nearly to drive her to distraction. I saw her in one of these attacks in bed with her entire surface covered and her eyes closed with œdema of the lids. The hives were so confluent that not a spot of healthy integument could be seen. The whole paroxysm lasted twenty-four hours. She seemed to be in terrible distress and exclaimed every moment, "I shall die this time surely!" She seemed suffocating and was throwing off the covers. It seemed from her movements and speech that her skin felt as if on fire. There was no perceptible thirst and time was precious, and I am satisfied that I made waste by my haste in giving her a dose of Apis$^{200}$, which had no effect. But the paroxysm passed off and another year rolled by, when she called on me, as I requested her to do, a month before the expected paroxysm. I then learned more of her symptoms. I learned that when the eruption was out distinctly in nearly all of the attacks she had found that heat calmed her terrible distress and ameliorated the itching and burning. While she craved cold and had even thrown the covers off she was made worse by it, but when she had retained presence of mind and covered herself warmly with clothing she soon became quiet and the paroxysm terminated with less suffering.

This being the case, Apis could not be her simillimum, and I could now understand clearly why I had failed to interrupt the paroxysm and bring about a feeling of contentment so usual in such cases. I have quieted such patients very frequently in an hour, and plainly as a result of a homœopathic remedy, but this case furnished me no evidence of curative action of my selected remedy. With the symptoms as given and the new modality, I gave her one dose of Rhus rad.$^{200}$, and bided my time ten days before the expected paroxysm. Within a few hours after taking the remedy she declared that her "spell" was coming on; but it was only the shadow, the paroxysm never appeared again. She has missed it two years and she is in better health than ever. She remarked to me one day, "Doctor, your powders have made a new woman of me." She had been treated allopathically, physiologically, eclectically, pathologically, and with all very badly. This may not have been urticaria. Some of the wise heads of the old school told her it was from eating strawberries, and she refrained from these luxurious fellows and still she did not miss the paroxysm. One told her one thing and another disputed him. What was it? I don't know, neither do I care. Perhaps some pathologist could inform me as to the *scientificity* of my prescription. I simply know that when comparing the pathogenesis found in the *Symptomen Codex* I found a picture of the disease to be cured, and that is enough for me. The highest potency at hand was administered and never repeated. The slight aggravation usual to such work followed, and then I was contented to await results.

I am contented with such results, and so will any man who knows how to apply the *law—the simillimum, the smallest dose, the dynamized drug.* In this way only can we progress, and in this way shall we become the most useful to our patrons.

